# Evaluation of the *EGFR* polymorphism R497K in two cohorts of neoadjuvantly treated breast cancer patients

**DOI:** 10.1371/journal.pone.0189750

**Published:** 2017-12-21

**Authors:** Marcelo Sobral-Leite, Esther H. Lips, Hayra de Andrade Vieira-Monteiro, Letícia Carlos Giacomin, Daniely Regina Freitas-Alves, Sten Cornelissen, Lennart Mulder, Jelle Wesseling, Marjanka K. Schmidt, Rosane Vianna-Jorge

**Affiliations:** 1 Division of Molecular Pathology, Netherlands Cancer Institute, Amsterdam, The Netherlands; 2 Coordenação de Pesquisa, Instituto Nacional de Câncer, Rio de Janeiro, RJ, Brasil; 3 Programa de Pós-Graduação em Saúde Pública e Meio Ambiente, Escola Nacional de Saúde Pública—FIOCRUZ, Rio de Janeiro, RJ, Brasil; 4 Division of Pathology, Netherlands Cancer Institute, Amsterdam, The Netherlands; 5 Programa de Farmacologia e Inflamação–Instituto de Ciências Biomédicas, Universidade Federal do Rio de Janeiro, Rio de Janeiro, RJ, Brasil; University of North Carolina at Chapel Hill School of Medicine, UNITED STATES

## Abstract

Pathological response of breast cancer to neoadjuvant chemotherapy (NAC) presents great variability, and new prognostic biomarkers are needed. Our aim was to evaluate the association of the epidermal growth factor receptor gene (*EGFR*) polymorphism R497K (rs2227983) with prognostic features and clinical outcomes of breast cancer, including the pathological response to NAC and the recurrence-free survival (RFS). Tumoral complete response (tCR) was defined by no remaining invasive cancer in the excised breast, whereas pathological complete response (pCR) was defined by no remaining invasive cancer both in the excised breast and lymph nodes. Two independent cohorts were analyzed: one from Brazil (INCA, n = 288) and one from The Netherlands (NKI-AVL, n = 255). In the INCA cohort, the variant (Lys-containing) genotypes were significantly associated with lower proportion of tCR (OR_adj_ = 0.92; 95%CI = 0.85–0.99), whereas in the NKI-AVL cohort they were associated with tumor grade 3 (p = 0.035) and with triple-negative subtype (p = 0.032), but not with clinical outcomes. Such distinct prognostic associations may have arisen due to different neoadjuvant protocols (p < 0.001), or to lower age at diagnosis (p < 0.001) and higher proportion of tumor grade 3 (p = 0.018) at the NKI-AVL cohort. Moreover, NKI-AVL patients achieved better proportion of pCR (21.2% *vs* 8.3%, p < 0.001) and better RFS (HR_adj_ = 0.48; 95% _adj_CI = 0.26–0.86) than patients from INCA. In conclusion, large scale studies comprehending different populations are needed to evaluate the impact of genome variants on breast cancer outcomes.

## Introduction

Breast cancer (BC) is the most frequent type of cancer in women both in the developed and the developing world [1]. It is a very heterogeneous disease with regards to its morphology, molecular profile, and clinical course [2], and new prognostic biomarkers are needed to improve treatment selection [3].

The epidermal growth factor receptor (EGFR) is a transmembrane tyrosine kinase receptor of the ErbB family, whose activation leads to mitogenic signaling (4). EGFR overexpression in BC is associated with large tumor size, poor differentiation, and poor clinical outcomes [4, 5]. Responses to anti-EGFR therapies in BC are suboptimal [6, 7], and it is possible that genetic variations affecting EGFR expression or signaling may contribute to the variability in treatment response [8].

The *EGFR* gene, located at 7p12.3-p.1, is rarely mutated in breast tumors [9], but harbors multiple polymorphisms. Among them, R497K (rs2227983) is a single nucleotide polymorphism (SNP), with a G→A change in exon 13 leading to an Arginine (Arg)→Lysine (Lys) substitution in codon 497. Moriai et al. showed an attenuated tyrosine kinase activity associated with the variant *'A'* allele, with consequent reductions in ligand binding, growth stimulation, and induction of proto-oncogenes *MYC*, *FOS*, and *JUN* [10]. Our group and Kallel et al. reported that BC patients carrying the variant R497K allele less often presented with lymph node metastasis than wild-type homozygous [11, 12]. Although the R497K-Lys variant seems to have no impact on the risk of developing cancer [13], it has been associated with better clinical outcomes in different cancer populations [14–22].

Over the past few years, neoadjuvant chemotherapy (NAC) has been used to downstage large tumors for breast surgery. As an additional contribution, patients who achieve pathological complete response (pCR) after NAC show better survival than those with partial response, depending on the subtype [23]. The individual response to NAC appears to be mostly dependent on the tumor molecular profile [24]: tumors lacking hormone receptors are more sensitive to cytotoxic chemotherapy [25], and, within triple-negative tumors, those overexpressing EGFR are the most responsive [26]. However, the great interpatient variability in the response to NAC cannot be completely explained by the molecular expression profile of breast tumors [27], which might be due to genetic variability [28].

In the present work, we aimed to characterize the potential contribution of R497K as a new biomarker in BC, by evaluating its association with histopathological features and with clinical outcomes of BC patients treated with NAC. Two independent cohorts of neoadjuvantly treated BC patients from different origins, *i*.*e*. Brazil and the Netherlands, were analyzed and compared.

## Materials and methods

### Study population

The study protocols followed the international precepts of ethics in research and of good clinical practice. The Ethics Committee of the Brazilian National Cancer Institute (INCA #129/08) and the ‘Protocol Toetsings Commissie’ of The Netherlands Cancer Institute-Antoni van Leeuwenhoek Hospital approved the study protocols. Informed consent was obtained from all subjects. The REMARK guidelines (REporting recommendations for tumor MARKer prognostic studies) were followed [31].

The study population was composed from two prospective cohorts of women with first diagnosis of unilateral BC, with no distant metastases, who were treated with NAC, in two national cancer centers, one in Brazil and one in the Netherlands. The Brazilian cohort (n = 325) was recruited at the Brazilian National Cancer Institute (INCA), at Rio de Janeiro (Brazil) during the period from February 2009 to April 2013, as described previously [29]. The Dutch cohort was formed at the Netherlands Cancer Institute—Antoni van Leeuwenhoek Hospital (NKI-AVL), in Amsterdam (The Netherlands), during the period from January 2004 to December 2015 (n = 1214). Eligibility for NAC at the NKI-AVL cohort was described previously [25, 30]. For the present study, only patients who had available DNA were included. At the NKI-AVL, DNA was collected only between 2007 and 2010 (n = 288). Also, the current analyses were restricted to patients who were submitted to surgery (either mastectomy or lumpectomy) after NAC. Therefore, the final study population was composed of 288 patients from INCA and 255 patients from the NKI-AVL.

### Clinical and histopathological data

Clinical and histopathological evaluations were performed according to institutional routine procedures, and all individual data were obtained from electronic medical records. The histopathological characterization of tumor biopsies from both cohorts followed the TNM classification by the American Joint Committee on Cancer [32] and on the Elston Ellis histological grading system [33]. Samples were considered positive for estrogen receptor (ER) or progesterone receptor (PR) when the staining was equal or higher than 1% of the tumor area. Because the standard cut-off value to define ER and PR positivity in NKI-AVL is 10% staining [34], all scores from NKI-AVL were reviewed, with tumors being reclassified as positive if the staining was at least 1% of the tumor area. Only 8 tumors (3.1%) had their status modified from negative to positive.

When HER2 expression was scored as 2+ or 3+ (on a scale of 0, 1+, 2+, or 3+), the number of copies of the HER2 gene was centrally determined by means of chromogenic in situ hybridization (CISH), when the material was available. BC subtypes were defined following previous studies [35]: luminal A (ER+ and/or PR+ and HER2-); luminal B (ER+ and/or PR+ and HER2+); HER2-like (ER-, PR- and HER2+); triple-negative (ER-, PR- and HER2-).

Tumoral complete response (tCR) was defined by the absence of remaining tumor or only *in situ* component after the neoadjuvant therapy (ypT0/is), whereas pathological complete response (pCR) was defined by tCR and no remaining tumor in axillary lymph nodes (ypT0/is ypN0) [23].

### NAC protocols

The NAC regimens were chosen by each institutional medical staff, according to local standard protocols or ongoing clinical trials.

Patients from the INCA cohort were assigned to receive one of the following protocols: 1.) three courses of 5-fluorouracil, doxorubicin and cyclophosphamide (FAC), followed by three courses of docetaxel (D); 2.) six courses of FAC or 3.) six courses of D. Few patients diagnosed with triple-negative tumors received six courses of cisplatin, doxorubicin and cyclophosphamide. All patients with HER2+ tumors were additionally treated with trastuzumab, which was initiated in combination with NAC protocols. After surgery, local radiotherapy was prescribed at medical discretion (68.8% received it), and patients with hormone receptor positive tumors also had subsequent endocrine therapy.

Patients from the NKI-AVL cohort were assigned for NAC depending on the particular study [25]. HER2-negative tumors were treated with one of the following: 1.) six courses of dose-dense doxorubicin/cyclophosphamide (AC); 2.) six courses of capecitabine/docetaxel (CapD); 3.) if the therapy response was considered ‘‘unfavorable” by magnetic resonance imaging (MRI) evaluation after three courses, AC was changed to CapD or vice versa [36]. All HER2+ patients were treated by a regimen of three cycles of 8-weekly courses of paclitaxel, trastuzumab, and carboplatin. Hormone receptor positive patients received both chemotherapy and endocrine therapy.

Variation of the standard protocols were detected in both cohorts, including number and order of the courses or substitution of one or 2 compounds. The final therapy that each patient received was allocated in one of the 4 categories: 1.) anthracycline/taxane; 2.) anthracycline-based; 3.) taxane-based or 4.) cisplatin/anthracycline. The full description of prescribed protocols and the groups where they were allocated can be assessed in S1 Table.

### Genotyping

Genomic DNA was obtained from peripheral blood samples, using either the Blood Genomic Prep Mini Spin Kit (GE Healthcare, Buckinghamshire, UK), for INCA samples, or DNAzol (DNAzol, Thermo Fisher Scientific, Waltham, MA, USA) for NKI-AVL samples [37]. All assays were performed blinded to the study endpoint.

At the INCA study, genotyping analyses were performed by PCR-RFLP, as described previously [11]. The method was validated by direct sequencing of four samples of each genotype (S1 Fig). At the NKI-AVL study, the genotyping analyses were performed by validated TaqMan assay (VIC- and FAM-labeled) for R497K SNP detection (Assay ID: C__16170352_20) purchased from Applied Biosystems (Applied Biosystems, Foster City, CA, USA). Sixteen DNA samples from the INCA cohort (4 from each genotype) were also genotyped at NKI-AVL. All genotype results matched. The genotypic distribution used for further analyses was: Arg/Arg (reference homozygous), Arg/Lys (heterozygous) and Lys/Lys (variant homozygous).

### Statistical analyses

Association tests were performed for cases with complete data. Associations between genotypes and clinical or pathological parameters were assessed by the *Chi*-square or Fisher’s exact tests (P), or with nonparametric trend test across ordered groups (P_trend_). Odds ratios (OR) with their respective 95% confidence intervals (95%CI) were adjusted for independent variables (OR_adj_) by logistic regression models. Only variables that showed a statistically significant association with genotypic groups (at least in one cancer center) were included in multivariable logistic regression models.

Recurrence-free survival (RFS) was calculated from the date of diagnosis to the date of disease recurrence (loco-regional recurrence or distant metastasis) or to end of follow up (censoring). New primary cancer lesions or deaths by causes unrelated to disease progression were also censored. Kaplan-Meier curves and log hazard estimations were performed with time right-censored at 5 years. Cumulative survival distributions were compared by log-rank test. Hazard ratios (HRs) were estimated using Cox regression models, stratified by cancer centers when applicable. Multivariable Cox models were fitted including age at diagnosis as continuous variable, tumor grade (1, 2, or 3), tumor size (≤ 2cm or > 2cm), lymph node status (negative or positive), subtypes (luminal A, luminal B, HER-like and triple-negative), chemotherapy protocol (anthracycline/taxane, anthracycline, taxane, or cisplatin/anthracycline) as categorical covariates. The analyses were performed including the missing values in the model if their proportion was higher than 3% of the sample size. Follow-up data of the NKI-AVL cohort was updated after last publication [25].

All P-values reported are from two-sided tests. The threshold for significance was set at P = 0.05 and highlighted in bold in Tables and figures. Statistical analyses were performed in R version 3.1.1.

## Results

The current study was conducted with patients who were submitted to surgery after NAC and had genotyping data (n = 288 for INCA and 255 for the NKI-AVL cohort). A flowchart describing the formation of each cohort in the study is shown in supplementary material (S2A Fig). The comparison of clinical-pathological variables at diagnosis between recruited and included patients of each cohort showed no significant differences, except for tumor size and lymph node status at the NKI-AVL cohort (S2 Table).

### Distribution of R497K genotypes and of clinical and histopathological features

The comparison of histopathological features between the two cohorts indicates significant differences in the proportions of lobular carcinomas and of grade 3 tumors, which were lower among INCA patients (Table 1). Regarding R497K, the variant Lys allele was more frequent at the NKI-AVL (0.25; 95%CI = 0.21–0.28) than at the INCA cohort (0.19; 95%CI = 0.16–0.23, p = 0.044), and the frequency of combined variant genotypes was slightly, yet significantly, higher in the NKI-AVL cohort (Table 1). The age at diagnosis also differed between the two cohorts (S2B Fig), being significantly lower at the NKI-AVL cohort (p < 0.001), with a median of 48 years-old (95%CI = 28–65), as compared with 52 years-old (95%CI = 28–72) at the INCA cohort.

**Table 1:** Histopathological characteristics, neoadjuvant protocols and R497K genotypes in INCA and NKI-AVL cohorts.

	Neoadjuvant series				
	INCA		NKI-AVL		
	*n*	%	*n*	%	P value^a^
Variables	288		255		
**Morphology**					**0.004**
ductal	269	93.4	174	86.6	
lobular	16	5.6	27	13.4	
others	3	1.0	0		
missing			54		
**Grade**					**0.018**
1	18	9.9	3	2.4	
2	115	63.2	78	61.9	
3	49	26.9	45	35.7	
missing	106		129		
**Tumor size**					0.184
T1/T2	170	59.6	165	65.2	
T3/T4	115	40.4	88	34.8	
missing	3		2		
**Lymph node status**					0.673
negative	63	21.9	52	20.4	
positive	225	78.1	203	79.6	
**ER status**					0.234
negative	85	29.6	87	34.8	
positive	202	70.4	163	65.2	
missing	1		5		
**PR status**					0.067
negative	132	46.2	132	54.5	
positive	154	53.8	110	45.5	
missing	2		13		
**HER2 status**					0.555
negative	215	78.8	189	76.2	
positive	58	21.2	59	23.8	
missing	15		7		
**Subtype**^**b**^					0.535
luminal A	160	58.6	129	52.2	
luminal B	32	11.7	33	13.4	
HER2-like	26	9.5	26	10.5	
triple negative	55	20.1	59	23.9	
missing	15		8		
**Chemotherapy**^**c**^					**<0.001**
anthracycline/taxane	271	94.1	51	20.0	
anthracycline	6	2.1	141	55.3	
taxane	6	2.1	63	24.7	
cisplatin/anthracycline	5	1.7	0		
**tCR**					**<0.001**
no	252	87.5	183	71.8	
yes	36	12.5	72	28.2	
**pCR**					**<0.001**
no	264	91.7	201	78.8	
yes	24	8.3	54	21.2	
**R497K**					0.137
Arg/Arg	192	66.7	149	58.4	
Arg/Lys	80	27.8	87	34.1	
Lys/Lys	16	5.6	19	7.5	
**R497K (Lys)**					**0.048**
Arg/Arg	192	66.7	149	58.4	
Arg/Lys + Lys/Lys	96	33.3	106	41.6	

(a) P value of the comparison between the two hospitals for each clinical variable. Missing data was not included in the P value calculation;

(b) Subtypes were defined as follows: Luminal A (ER+ and/or PR+ and HER2-); Luminal B (ER+ and/or PR+ and HER2+); HER2-like (ER-, PR- and HER2+); Triple negative (ER-, PR- and HER2-).

(c) Patients with HER2+ tumors in both centers received trastuzumab (not shown in the table). Abbreviations: complete tumor response (tCR), pathologic complete response (pCR).

The cohorts also differed regarding NAC regimens (Table 1): almost all INCA patients received a combination of anthracycline and taxane, which is the institutional standard protocol, whereas NKI-AVL patients were most commonly treated with anthracycline-based combinations in the initial cycles of chemotherapy, with a possibility of switching to taxane if their middle-course response was evaluated as unfavorable by MRI monitoring. A full description of all chemotherapeutic protocols used in both institutions is presented in S1 Table.

Regarding treatment outcomes, patients from the NKI-AVL cohort showed better pathological response after NAC (both tCR and pCR) than patients from INCA (Table 1). The two cohorts also differed in relation to RFS curves (p < 0.001), with a favorable profile for NKI-AVL in comparison to INCA (S2C Fig).

Table 2 presents the distribution of R497K genotypes according to clinical and histopathological categories. The results show statistically different distributions of R497K genotypes according to grade (p = 0.035) and tumor subtype (p = 0.032), only in NKI-AVL cohort, with the variant (Lys-containing) genotypes being significantly associated with grade 3 and with triple-negative tumors.

**Table 2:** Distribution of clinical and histopathological variables according to R497K genotypes in INCA and NKI-AVL cohorts.

	INCA					NKI-AVL					
	Arg/Arg		Arg/Lys + Lys/Lys			Arg/Arg		Arg/Lys + Lys/Lys			
Variables	*n*	%	*n*	%	P value^a^	*n*	%	*n*	%	P value^a^	P value^b^
**Grade**					0.106					**0.035**	0.081
1	9	7.4	9	15.0		1	1.3	2	4.2		
2	83	68.0	32	53.3		55	70.5	23	47.9		
3	30	24.6	19	31.7		22	28.2	23	47.9		
missing	70		36			71		58			
**Tumor size**					1.000					1.000	0.522
T1/T2	113	59.8	57	59.4		97	65.5	68	64.8		
T3/T4	76	40.2	39	40.6		51	34.5	37	35.2		
missing	3		0			1		1			
**Lymph node status**					0.880					0.054	0.154
negative	41	21.4	22	22.9		37	24.8	15	14.2		
positive	151	78.7	74	77.1		112	75.2	91	85.9		
**ER status**					0.068					0.089	**0.006**
negative	64	33.3	21	22.1		44	30.1	43	41.4		
positive	128	66.7	74	77.9		102	69.9	61	58.7		
**PR status**					0.064					0.528	**0.010**
negative	96	50.3	36	37.9		74	52.5	58	57.4		
positive	95	49.7	59	62.1		67	47.5	43	42.6		
**HER2 status**					0.340					0.130	0.996
negative	139	76.8	76	82.6		105	72.4	84	81.6		
positive	42	23.2	16	17.4		40	27.6	19	18.4		
missing	11		4			4		3			
**Subtype**					0.129					**0.032**	**0.019**
luminal A	100	55.3	60	65.2		76	52.8	53	51.5		
luminal B	20	11.1	12	13.0		26	18.1	7	6.8		
HER2-like	22	12.2	4	4.4		14	9.7	12	11.7		
triple negative	39	21.6	16	17.4		28	19.4	31	30.1		
missing	11		4			5		3			
**Chemotherapy**					0.338					0.455	**<0.001**
anthracycline/taxane	178	92.7	93	96.9		28	18.8	23	21.7		
anthracycline	5	2.6	1	1.0		80	53.7	61	57.6		
taxane	4	2.1	2	2.1		41	27.5	22	20.8		
cisplatin/anthracycline	5	2.6	0	0.0				0	0.0		
**tCR**					**0.005**					0.455	**<0.001**
no	160	83.3	92	95.8		109	73.2	74	69.8		
yes	32	16.7	4	4.2		40	26.9	32	30.2		
**pCR**					**0.013**					0.987	**<0.001**
no	170	88.5	94	97.9		118	79.2	83	78.3		
yes	22	11.5	2	2.1		31	20.8	23	21.7		

(a) P value of the distribution comparison of the clinical variables by the genotype groups.

(b) P value of the distribution comparison of the variant genotype groups by the cancer centers. Abbreviations: complete tumor response (tCR), pathologic complete response (pCR).

### R497K and the pathological response to NAC

The data presented in Table 2 indicate significantly different proportions of tCR and pCR according to R497K genotypes within the INCA cohort, but not among patients from the NKI-AVL. After multivariable analysis (Table 3), R497K variant genotypes maintained the significant association with tCR, with patients carrying at least one variant allele (Arg/Lys or Lys/Lys) being less likely to achieve tCR than those with the Arg/Arg genotype. Again, no significant associations between R497K genotypes and pathological response were observed in the NKI-AVL cohort. As expected, HER2-like and triple-negative subtypes were associated with pCR in both cohorts, but not with R497K genotype groups (Table 3).

**Table 3:** Impact of R497K polymorphism and other variables on the response to neoadjuvant chemotherapy.

	Multivariable model for neoadjuvant chemotherapy response (tCR and pCR)																																			
	INCA												NKI-AVL												INCA + NKI-AVL											
	tCR						pCR						tCR						pCR						tCR						pCR					
Variables	OR_adj_^a^		CI (95%)				OR_adj_^a^		CI (95%)				OR_adj_^a^		CI (95%)				OR_adj_^a^		CI (95%)				OR_adj_^a^		CI (95%)				OR_adj_^a^		CI (95%)			
**R497K genotype**																																				
Arg/Arg	1.00						1.00						1.00						1.00						1.00						1.00					
Arg/Lys + Lys/Lys	**0.92**	**(**	**0.85**	**─**	**0.99**	**)**	0.95	(	0.89	─	1.01	)	1.04	(	0.93	─	1.15	)	1.03	(	0.93	─	1.13	)	0.97	(	0.91	─	1.04	)	0.98	(	0.93	─	1.04	)
**Subtype**																																				
luminal A	1.00						1.00						1.00						1.00						1.00						1.00					
luminal B	**1.13**	**(**	**1.00**	**─**	**1.28**	**)**	1.03	(	0.93	─	1.15	)	**1.69**	**(**	**1.28**	**─**	**2.23**	**)**	**1.57**	**(**	**1.22**	**─**	**2.03**	**)**	**1.24**	**(**	**1.11**	**─**	**1.39**	**)**	**1.14**	**(**	**1.04**	**─**	**1.27**	**)**
HER2-like	**1.19**	**(**	**1.04**	**─**	**1.36**	**)**	**1.22**	**(**	**1.09**	**─**	**1.37**	**)**	**1.92**	**(**	**1.46**	**─**	**2.51**	**)**	**1.83**	**(**	**1.43**	**─**	**2.34**	**)**	**1.38**	**(**	**1.22**	**─**	**1.55**	**)**	**1.36**	**(**	**1.22**	**─**	**1.51**	**)**
triple negative	**1.15**	**(**	**1.04**	**─**	**1.29**	**)**	**1.15**	**(**	**1.05**	**─**	**1.26**	**)**	**1.33**	**(**	**1.16**	**─**	**1.52**	**)**	**1.25**	**(**	**1.11**	**─**	**1.42**	**)**	**1.26**	**(**	**1.16**	**─**	**1.37**	**)**	**1.21**	**(**	**1.13**	**─**	**1.31**	**)**
missing	1.12	(	0.95	─	1.33	)	1.09	(	0.95	─	1.26	)	1.19	(	0.87	─	1.62	)	1.22	(	0.91	─	1.62	)	1.14	(	0.97	─	1.34	)	1.13	(	0.98	─	1.30	)
**Chemotherapy**																																				
anthracycline/taxane	1.00						1.00						1.00						1.00						1.00						1.00					
anthracycline	1.02	(	0.79	─	1.32	)	1.04	(	0.84	─	1.28	)	1.13	(	0.99	─	1.29	)	1.08	(	0.95	─	1.22	)	1.07	(	0.96	─	1.19	)	1.03	(	0.94	─	1.13	)
taxane	0.92	(	0.71	─	1.19	)	0.95	(	0.76	─	1.17	)	0.99	(	0.77	─	1.28	)	0.98	(	0.78	─	1.23	)	**1.18**	**(**	**1.03**	**─**	**1.35**	**)**	**1.18**	**(**	**1.05**	**─**	**1.33**	**)**
cisplatin/anthracycline	**1.46**	**(**	**1.09**	**─**	**1.95**	**)**	**1.53**	**(**	**1.20**	**─**	**1.94**	**)**	(empty)						(empty)						**1.43**	**(**	**1.03**	**─**	**1.99**	**)**	**1.51**	**(**	**1.13**	**─**	**2.02**	**)**
**Cancer center**																																				
INCA																									1.00						1.00					
NKI-AVL																									1.06	(	0.96	─	1.17	)	1.07	(	0.98	─	1.17	)

(a) Multivariable models using logistic regression including the following variables: age at diagnosis, histological grade, tumor size, lymph node metastasis, tumor subtype, chemotherapy regiment and R497K genotype. Note: Only variables that showed a statistical association in one of the four models were plotted in the table. The reference groups used for OR association calculation in each variable is indicated with OR = 1.00. Cells were left empty when there were no observations in the group and omitted when there were not enough observations for calculations. Total observations included in the models: INCA cohort 285 and NKI-AVL cohort 253 observations (3 and 2 observations excluded due to missingness, respectively) Abbreviations: complete tumor response (tCR), pathologic complete response (pCR), adjusted OR (OR_adj_), confidence interval (CI).

Additionally, the association of R497K genotypes with pathological response was further evaluated in subsets of patients treated with similar NAC protocols (S3 Fig). At the INCA cohort, the anthracycline/taxane-based chemotherapy was majoritarian (94% of patients), and so it was the only subset analyzed. The results indicate a beneficial effect of variant genotypes both for tCR (OR = 0.89; 95%CI = 0.82–0.96) and for pCR (OR = 0.92; 95%CI = 0.86–0.99). At the NKI-AVL cohort, no differences in pathological response, either tCR or pCR, were observed according to R497K genotypes irrespective of which NAC protocol was used.

### Survival analysis according to R497K

**Fig 1:**
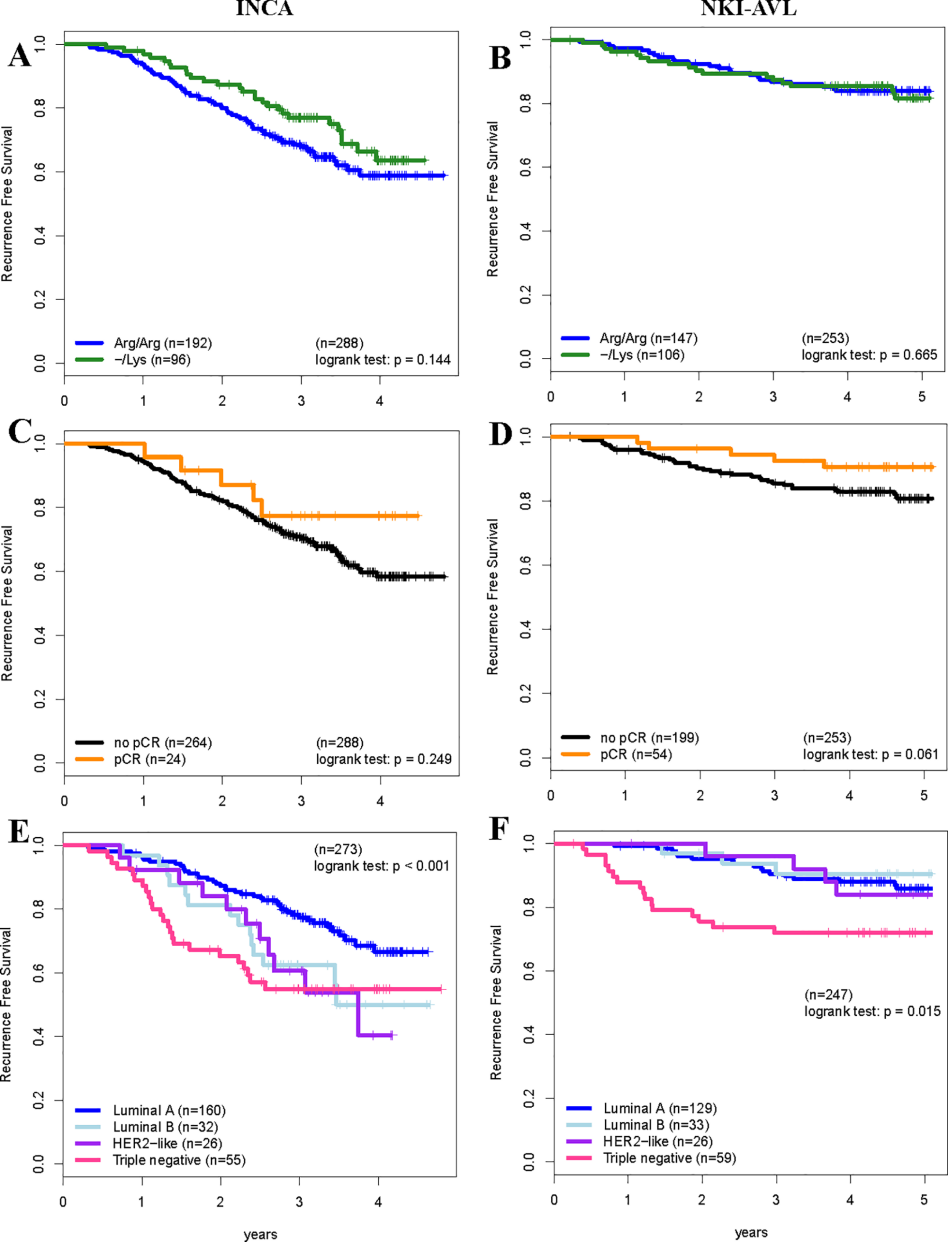
Survival analysis. Relapse free survival (RFS) curves according to R497K polymorphism groups (A, B), pathological complete response (pCR) (C, D) and BC subtypes (E, F) in patients from INCA cohort (A, C, E) and NKI-AVL cohort (B, D, F).

The median follow-up was 2.8 years for INCA and 4.8 years for the NKI-AVL cohort. Table 4 shows the impact of R497K and of clinical-pathological variables on RFS in the two cohorts, either considered separately or together. R497K showed no significant association with RFS in either cohort. Positive lymph node status and triple-negative subtype were the only predictors of worse RFS curves in both cohorts. Patients with pCR had better RFS in the INCA cohort, but not in the NKI-AVL cohort. Fig 1 shows the RFS curves according to R497K (Fig 1A and 1B), pCR (Fig 1C and 1D), or tumor subtype (Fig 1E and 1F).

**Table 4:** Proportional hazard models according to prognostic variables and R497K.

	Hazard ratios for 5 years RFS																	
	Multivariate analysis																	
	INCA						NKI-AVL						INCA+NKI-AVL					
Variables	HR_adj_^a^		CI (95%)				HR_adj_^a^		CI (95%)				HR_adj_^a^		CI (95%)			
**R497K genotype**																		
Arg/Arg	1.00						1.00						1.00					
Arg/Lys + Lys/Lys	0.69	(	0.43	─	1.10	)	1.01	(	0.55	─	1.86	)	0.81	(	0.56	─	1.16	)
**Age at diagnosis**																		
per year older	0.98	(	0.97	─	1.00	)	0.99	(	0.96	─	1.02	)	0.98	(	0.97	─	1.00	)
**Grade**																		
1 + 2	1.00						1.00						1.00					
3	1.04	(	0.58	─	1.86	)	1.46	(	0.64	─	3.35	)	1.15	(	0.73	─	1.83	)
missing	0.93	(	0.57	─	1.52	)	0.59	(	0.28	─	1.23	)	0.80	(	0.54	─	1.20	)
**Tumor size**																		
T1/T2	1.00						1.00						1.00					
T3/T4	**2.08**	**(**	**1.35**	**─**	**3.21**	**)**	1.32	(	0.72	─	2.45	)	**1.77**	**(**	**1.26**	**─**	**2.49**	**)**
**Lymph node status**																		
negative	1.00						1.00						1.00					
positive	**2.58**	**(**	**1.36**	**─**	**4.91**	**)**	**7.11**	**(**	**1.70**	**─**	**29.70**	**)**	**3.41**	**(**	**1.93**	**─**	**6.04**	**)**
**Subtype**																		
luminal A	1.00						1.00						1.00					
luminal B	1.42	(	0.74	─	2.71	)	0.62	(	0.14	─	2.79	)	1.34	(	0.75	─	2.39	)
HER2-like	1.81	(	0.86	─	3.81	)	0.81	(	0.17	─	3.81	)	**1.90**	**(**	**1.02**	**─**	**3.53**	**)**
triple negative	**2.85**	**(**	**1.59**	**─**	**5.12**	**)**	**3.64**	**(**	**1.75**	**─**	**7.58**	**)**	**3.14**	**(**	**2.00**	**─**	**4.92**	**)**
missing	0.93	(	0.29	─	3.06	)	2.01	(	0.23	─	17.58	)	1.17	(	0.42	─	3.28	)
**Chemotherapy**																		
anthracycline/taxane	1.00						1.00						1.00					
anthracycline	1.22	(	0.33	─	4.42	)	0.44	(	0.21	─	0.95	)	0.69	(	0.36	─	1.33	)
taxane	0.97	(	0.23	─	4.09	)	1.03	(	0.28	─	3.72	)	0.78	(	0.34	─	1.76	)
cisplatin/anthracycline	1.39	(	0.31	─	6.15	)							1.30	(	0.30	─	5.56	)
**pCR**																		
no	1.00						1.00						1.00					
yes	**0.24**	**(**	**0.08**	**─**	**0.70**	**)**	0.37	(	0.14	─	1.02	)	**0.28**	**(**	**0.14**	**─**	**0.57**	**)**
**Cancer center**																		
INCA													1.00					
NKI-AVL													**0.48**	**(**	**0.26**	**─**	**0.86**	**)**

(a) Hazard ratios were adjusted with the variables that were significantly associated with RFS in the univariate analysis. The reference groups used for HR risk calculation in each variable is indicated with HR = 1.00. Cells were left empty when there were no observations in the group and omitted when there were not enough observations for the model calculations. Total observations included in the models: INCA cohort 285 and NKI-AVL cohort 251 observations (3 and 4 observations excluded due to missingness, respectively). Abbreviations: relapse free survival (RFS), pathologic complete response (pCR), hazard ratio (HR), adjusted hazard ratio (HR_adj_), confidence interval (CI).

When the two cohorts were merged for analysis, the cancer center also showed strong association with RFS; patients treated at NKI-AVL showed better RFS compared with those treated at INCA (HR_adj_ = 0.48; 95% _adj_CI = 0.26–0.86; Table 4 and S2C Fig).

## Discussion

The present work aimed to evaluate the possible contribution of *EGFR* R497K as a biomarker in BC, especially regarding its possible impact on clinical outcomes, such as the pathological response to NAC and the subsequent RFS. The analysis was first conducted within a Brazilian cohort of BC patients treated with NAC; hereafter, an independent cohort of neoadjuvantly treated BC patients in the Netherlands was analyzed, and results of both cohorts were compared. Such comparison was not meant as a strict validation step, since the two cohorts are not alike regarding patients’ origin and specific clinical conducts. However, to the best of our knowledge, there are not many cohorts of neaoadjuvantly treated breast cancer patients with available DNA or genotyping data, as well as complete information on chemotherapeutic protocols and patients’ follow-up. Therefore, it seemed a good opportunity to compare these two cohorts, which, despite specific aspects, include current standard protocols of tumor classification and neoadjuvant treatment of breast cancer.

The two cohorts were mostly similar regarding histopathological and clinical features at diagnosis, with differences only in grade distribution and in morphology, with higher frequency of lobular tumors among NKI-AVL patients. Despite the significant difference, the frequencies of lobular tumors in both cohorts are within the expected incidence of 5–15% [38, 39]. Regarding R497K, the variant Lys allele was apparently more frequent in the NKI-AVL than in the INCA cohort, although such frequencies are not significantly different from previously reported for another INCA cohort, composed mostly of early-stage BC patients treated with curative surgery as first therapeutic approach [11].

The comparison of NAC regimens between the two cohorts indicates a greater variety among NKI-AVL protocols, which also included capecitabine. Nevertheless, all protocols used, even those including capecitabine, are usually considered equivalent in their efficacy [40].

In view of the similar histopathological characteristics and equivalent pharmacological approach of the two cohorts, the lower proportion of pCR and tCR at INCA was not expected, and is quite challenging. Maybe the NKI-AVL strategy of monitoring NAC response using MRI applied on ER-positive, HER2-negative patients, and changing protocols in cases of limited response may explain the better ratio of NAC responses in NKI-AVL, compared with INCA cohort [36]. The impact of MRI monitoring on NAC responses is reflected by the differences in tCR and pCR rates among groups receiving different NAC protocols at NKI-AVL cohort (S3 Fig). Nevertheless, when the two cohorts were merged for analysis, the cancer center was not significantly associated with the response rates to NAC (Table 3). In fact, the only independent predictor of tCR or pCR in the two cohorts, analyzed either separately or together, was tumor subtype, with luminal tumors being the least responsive, whereas triple-negative tumors had the best response rates. This latter result is in accordance with extensive data in the literature, which indicate that triple-negative tumors are the most responsive to NAC [25], although they show worse prognosis with regards to RFS [41]. This apparently contradictory profile may be explained by the highly proliferative pattern of triple-negative tumors [42].

Finally, regarding R497K, the results suggest a possible weak association between R497K variant genotypes and reduced NAC response, which was observed only at the INCA cohort. Although such association was rejected after multivariable analysis, the low rate of pCR in the INCA cohort might have limited the power for the statistical analysis, possibly leading to a type II error (failure to reject a false null hypothesis). Previous reports have shown that R497K Lys-variants may attenuate ligand binding and tyrosine kinase activation, leading to lower cellular growth, migration and proliferation [10], which could explain a lower rate of tCR and pCR compared with the wild-type (Arg/Arg) genotype.

The apparent association between R497K and the response rates to NAC at INCA was not observed in the NKI-AVL patients, which presented better response rates. One possible reason for this lack of effect for R497K at the NKI-AVL cohort is the higher frequency of the variant (Lys-containing) genotypes among grade 3 and among triple-negative tumors, which are more responsive to NAC. This association, which was not seen at the INCA cohort, may have superposed any potential effect of R497K, and interfered with its use as a predictor of the tumor response to NAC.

The differences between the two cohorts regarding the association of R497K with histopathological features of BC and with its response to NAC illustrate how the prognostic effects of specific polymorphism may vary according to the populations. The confirmation of a possible negative effect of variant R497K in the response to NAC chemotherapy would require larger cohorts of BC patients, ideally under the same treatment conditions.

At last, we analyzed the possible impact of R497K on RFS, with no significant association being found in either cohort. Although the two cohorts were similar in the lack of R497K effect, they had significantly different RFS, with the NKI-AVL presenting better profile than INCA. Such difference was not to be expected, considering that the two cohorts had similar tumor staging and molecular profile at diagnosis. Also, despite the larger diversity of NAC protocols at the NKI-AVL, the pharmacological combinations available in both institutions are considered equivalent in their efficacy [40].

The evaluation of which variables were affecting RFS indicated that high tumor sizes and pCR were significantly associated with worse or better RFS, respectively, but only at the INCA cohort. The lack of such associations within the NKI-AVL cohort might be due to the better NAC responses rates if compared to INCA.Regarding NAC protocols, the anthracycline-based therapy had an apparent beneficial effect on RFS if compared to anthracycline-taxane combinations in the NKI-AVL cohort. This apparent difference, however, reflects the selective use of the latter protocol for patients whose partial response at middle course of NAC regimen was considered insufficient.

In addition to possible differences in neoadjuvant conducts affecting RFS, the two centers also differed regarding surgical procedures and adjuvant protocols. Mastectomy was the standard surgical strategy at INCA (only 3 patients had breast-conserving surgery), whereas the proportion of mastectomy and lumpectomy at the NKI-AVL was about fifty-fifty. The available literature does not favor mastectomy over breast-conserving procedures with regards to recurrence-free or overall survival in patients with good or partial response to NAC [43, 44]. However, it is also unlikely that the higher proportion of lumpectomies within the NKI-AVL would favor longer RFS [44]. The use of adjuvant radiotherapy within the INCA cohort did not affect RFS (P_Log-Rank_ = 0.149), but it is unknown if the variability of adjuvant chemotherapy protocols has contributed for the differences in RFS between the two cohorts.

Survival in BC may also be affected by health disparities in cancer within and across countries [45]. A comparison of BC presentation and outcome among countries of middle and high income is described by Saxena et al [46]. In Asia, despite small differences in prognostic factors at presentation, overall survival of BC patients from Malaysia was much lower than that of Singaporean patients (both middle income countries, like Brazil (http://www.who.int/countries). Thus, large-scale studies on pharmacogenetic analyses are required to understand how polymorphisms affecting the EGFR/PI3K/PTEN/Akt/mTOR pathway could open possibilities for pharmacotherapy precision in BC [8, 47].

## Conclusion

The current results suggest that R497K polymorphism might be associated with NAC resistance in specific populations or treatment conditions. From a global perspective, the clinical inter-population variability stressed in this study highlight the challenge on demonstrating the impact of genome variants on cancer outcomes.

## Supporting information

S1 Table Characterization of neoadjuvant protocols at INCA and NKI-AVL breast cancer cohorts included in the study. Description of the complete cohort, from both cancer centers: INCA and NKI-AVL.
(PDF)

S2 Table Characterization of the sub-cohort used for association analyses. Comparison of the clinical and pathological features between the analyzed group and the complete cohorts, from both cancer centers: INCA and NKI-AVL.
(PDF)

S1 Fig R497K genotyping. Representative PCR-RFLP patterns of rs2227983 on 2% agarose gel. Genomic DNA was used on PCR amplification of exon 13 using 5’-AGGTCTGCCATGCCTTGT-3’ (forward) and 5’- CAACGCAAGGGGATTAAAGA-3’ (reverse) and then digested by BstN1 restriction enzyme at 60─C for 3 h (A). Direct sequencing results of rs2227983 in Applied Biosystems Prism 3130 genetic analyzer. The sequence graphs of the wild type (Arg/Arg—GG), the heterozygous (Arg/Lys—GA) and the homozygous variant (Lys/Lys–AA) are represented in colors (B). Abbreviation: control without digestion: “C-─.
(PDF)

S2 Fig Data collection. Flow chart describing the formation of each cohort from patients originally admitted in the two cancer centers: INCA and NKI-AVL (A). Distribution of the age at diagnosis of included patients from each cohorts (B). Relapse free survival curves of the breast cancer patients included in the study according to the cancer center where they were treated; 2 observations from the NKI-AVL cohort were not included in the plot due to missing follow-up data (C). (*) Genome DNA was only available for patients included in the NKI-AVL cohort between 2007 and 2010.
(PDF)

S3 Fig Pathological response according to R497K genotypes within similar NAC protocols. Proportions of cases who achieved tumor complete response (tCR) according to R497K genotypes after neoadjuvant treatment at INCA based on anthracycline/taxane chemotherapy protocols (A) or at NKI-AVL based on anthracycline/taxane (B), anthracycline (C) or taxane (D) protocols. In panels E-H, the same subgroup analysis comparing the proportion of pathological complete response status (pCR) at INCA (E) and NKI-AVL (F-H) cohorts. Numbers in parenthesis correspond to the total number of cases in each genotype group. P values were assessed by the Chi-square, or Fisher’s exact tests when counts were equal zero in at least one group. At the INCA cohort, the anthracycline/taxane protocol was majoritarian (94%) and, therefore, the sample size of other chemotherapy protocols was insufficient for comparisons.
(PDF)

S1 File The REMARK checklist. List of guidelines from the Recommendations for Tumor Marker Prognostic Studies (REMARK) that were used in this study.
(PDF)
