# Supplementary material for: Evaluation of the EGFR polymorphism R497K in two cohorts of neoadjuvantly treated breast cancer patients
Source: PLoS One. 2017 Dec 21;12(12):e0189750. doi: 10.1371/journal.pone.0189750 (PMC5739423; doi:10.1371/journal.pone.0189750)
Supplement: S1 Table — Description of the complete cohort, from both cancer centers: INCA and NKI-AVL. (PDF) [file pone.0189750.s001.pdf]

	**INCA**			**NKI-AVL**	
	n	%		*n*	%
**Protocol type**	288	100.00	**Protocol type**	255	100.00
**anthracycline/ taxane**			**anthracycline/ taxane**		
3 FAC - 3 D	224	82.66	3 AC - 3 CapD	35	68.63
3 D - 3 FAC	18	6.64	3 AC - 1 CapD	3	5.88
6 FAC - 3 D	2	0.74	3 AC - 16 PacCarbo	2	3.92
3 FAC - 4 D	2	0.74	8 PacCarbo - 4 FEC	2	3.92
other combinations	25	9.23	3 AC - 2 CapD	2	3.92
total	*271*	*100.00*	other combinations	7	13.73
			total	*51*	*100.00*
**anthracycline**			**anthracycline**		
6 FAC	3	50.00	6 AC	125	88.65
4 FAC	2	33.33	4 AC - 2 CTCarbo	10	4.26
3 FAC	1	16.67	3 AC	4	2.84
total	*6*	*100.00*	other combinations	2	4.26
			total	*141*	*100.00*
**taxane**			**taxane**		
4 C + D	2	33.33	24 PacCarbo	55	87.30
4 D + C	2	33.33	6 CapD	4	6.35
3 D - 4 C + D	1	16.67	8 PacCarbo	3	4.76
2D	1	16.67	5 CapD	1	1.59
total	*6*	*100.00*	*total*	*63*	*100.00*
**cisplatin/ anthracycline**			**cisplatin/ anthracycline**		
6 Cis + A + C	5	100.00		0	

Note: The categories "other combinations" comprehend small protocol modifications, such as variations in the number of chemotherapy courses, drug substitutions within the same pharmacological class or sequence inversions. Patients with HER2+ tumors in both centers received trastuzumab (not shown in the table). Abbreviations: 5-fluorouracil (F), doxorubicin (A), epirrubicin (E), cyclophosphamide (C), docetaxel (D), cisplatin (Cis), capecitabine (Cap), thiothepa (T), paclitaxel (Pac), carboplatin (Carbo), "followed by" ("-") and "together with" ("+"). Protocols: 5-fluorouracil + doxorubicin + cyclophosphamide (FAC), doxorubicin + cyclophosphamide (AC), capecitabine + docetaxel (CapD), paclitaxel + capecitabine (PacCarbo), 5-fluorouracil + epirrubicin + cyclophosphamide (FEC), cyclophosphamide + thiotepa + carboplatin (CTCarbo).
