# Supplementary material for: Evaluation of the EGFR polymorphism R497K in two cohorts of neoadjuvantly treated breast cancer patients
Source: PLoS One. 2017 Dec 21;12(12):e0189750. doi: 10.1371/journal.pone.0189750 (PMC5739423; doi:10.1371/journal.pone.0189750)
Supplement: S2 Table — Comparison of the clinical and pathological features between the analyzed group and the complete cohorts, from both cancer centers: INCA and NKI-AVL. (PDF) [file pone.0189750.s002.pdf]

	INCA					NKI-AVL				
	Recruited		Included			Recruited		Included		
	n	%	*n*	%	P value[a]	*n*	%	*n*	%	P value[a]
**Variables**	325		288			1214		255		
**Morphology**					0.973					0.335
ductal	305	93.8	269	93.4		546	89.1	174	86.6	
lobular	17	5.2	16	5.6		67	10.9	27	13.4	
others	3	0.9	3	1.0		0	0.0	0	0.0	
missing	0		0			601		54		
**Grade**					0.757					0.331
1	18	8.6	18	9.9		41	5.4	3	2.4	
2	129	61.4	115	63.2		443	58.5	78	61.9	
3	63	30.0	49	26.9		273	36.1	45	35.7	
missing	115		106			457		129		
**Tumor size**					0.758					**0.034**
T1/T2	184	58.4	170	59.6		504	72.3	165	65.2	
T3/T4	131	41.6	115	40.4		193	27.7	88	34.8	
missing	10		3			517		2		
**Lymph node status**					0.753					**0.001**
negative	70	22.3	59	21.2		220	31.3	52	20.4	
positive	244	77.7	219	78.8		483	68.7	203	79.6	
missing	11		10			511		0		
**ER status**					0.801					0.193
negative	99	30.6	85	29.6		357	39.3	87	34.8	
positive	225	69.4	202	70.4		551	60.7	163	65.2	
missing	1		1			306		5		
**HER2 status**					0.740					0.468
negative	246	79.9	215	78.8		710	78.4	189	76.2	
positive	62	20.1	58	21.2		196	21.6	59	23.8	
missing	17		15			308		7		
**pCR**					0.943					0.123
no	280	87.2	264	91.7		499	70.5	201	78.8	
yes	26	8.1	24	8.3		176	24.9	54	21.2	
no surgery	15	4.7				33	4.7			
missing	4					506				
**R497K**					0.982					
Arg/Arg	205	66.8	192	66.7				149	58.4	
Arg/Lys	86	28.0	80	27.8				87	34.1	
Lys/Lys	16	5.2	16	5.6				19	7.5	
missing	18							0		

(a) P value of the comparison between included cases and analyzed cases in each cohort. Missing data was not included in the P value calculation. Abbreviation: pathologic complete response (pCR).
