# Supplementary figures and images for: Evaluation of the EGFR polymorphism R497K in two cohorts of neoadjuvantly treated breast cancer patients
Source: PLoS One. 2017 Dec 21;12(12):e0189750. doi: 10.1371/journal.pone.0189750 (PMC5739423; doi:10.1371/journal.pone.0189750)

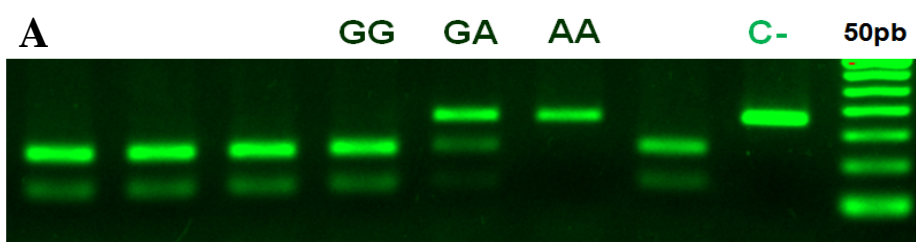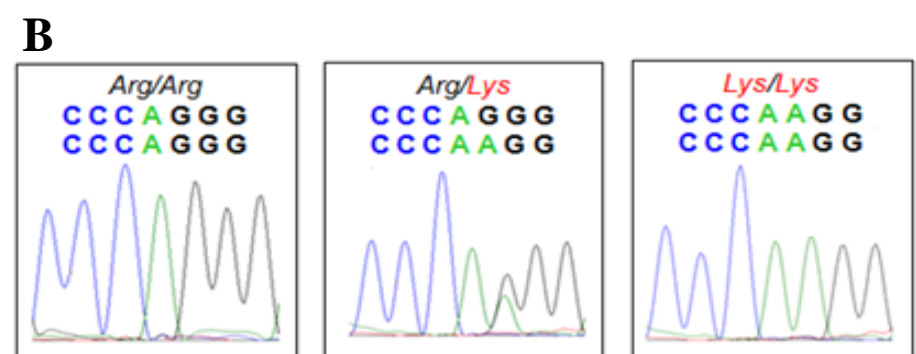

Supplement: S1 Fig — Representative PCR-RFLP patterns of rs2227983 on 2% agarose gel. Genomic DNA was used on PCR amplification of exon 13 using 5’-AGGTCTGCCATGCCTTGT-3’ (forward) and 5’- CAACGCAAGGGGATTAAAGA-3’ (reverse) and then digested by BstN1 restriction enzyme at 60─C for 3 h (A). Direct sequencing results of rs2227983 in Applied Biosystems Prism 3130 genetic analyzer. The sequence graphs of the wild type (Arg/Arg—GG), the heterozygous (Arg/Lys—GA) and the homozygous variant (Lys/Lys–AA) are represented in colors (B). Abbreviation: control without digestion: “C-─. (PDF) [file pone.0189750.s003.pdf]

**A**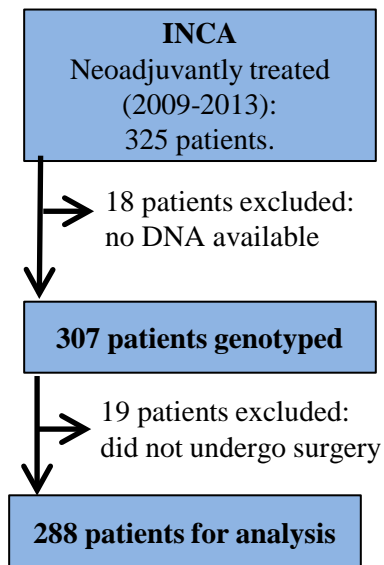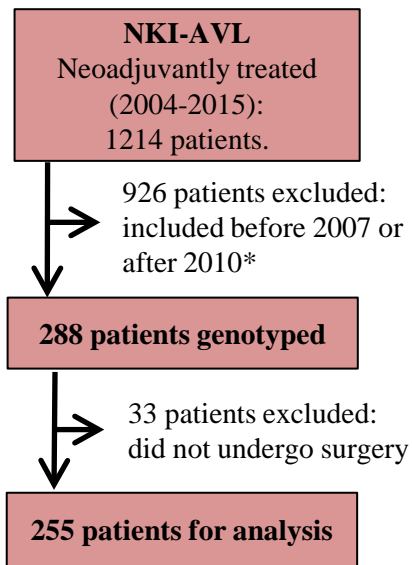**B**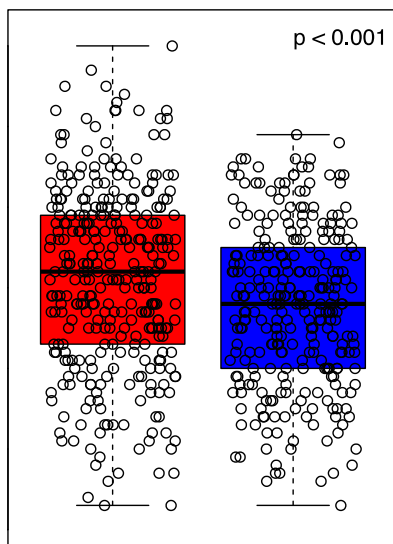**C**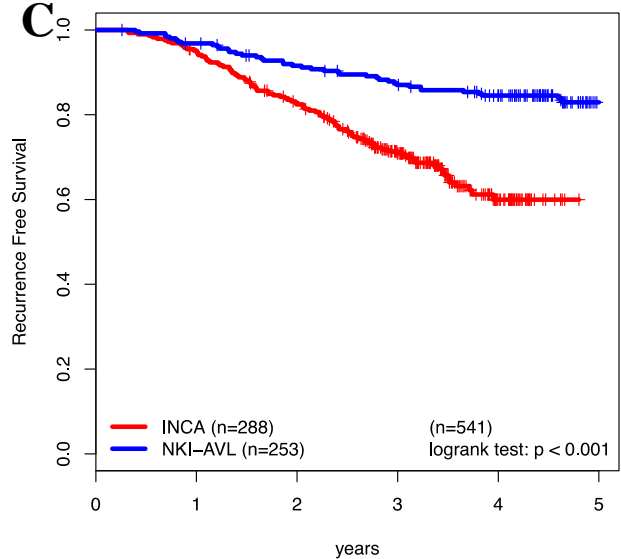

Supplement: S2 Fig — Flow chart describing the formation of each cohort from patients originally admitted in the two cancer centers: INCA and NKI-AVL (A). Distribution of the age at diagnosis of included patients from each cohorts (B). Relapse free survival curves of the breast cancer patients included in the study according to the cancer center where they were treated; 2 observations from the NKI-AVL cohort were not included in the plot due to missing follow-up data (C). (*) Genome DNA was only available for patients included in the NKI-AVL cohort between 2007 and 2010. (PDF) [file pone.0189750.s004.pdf]

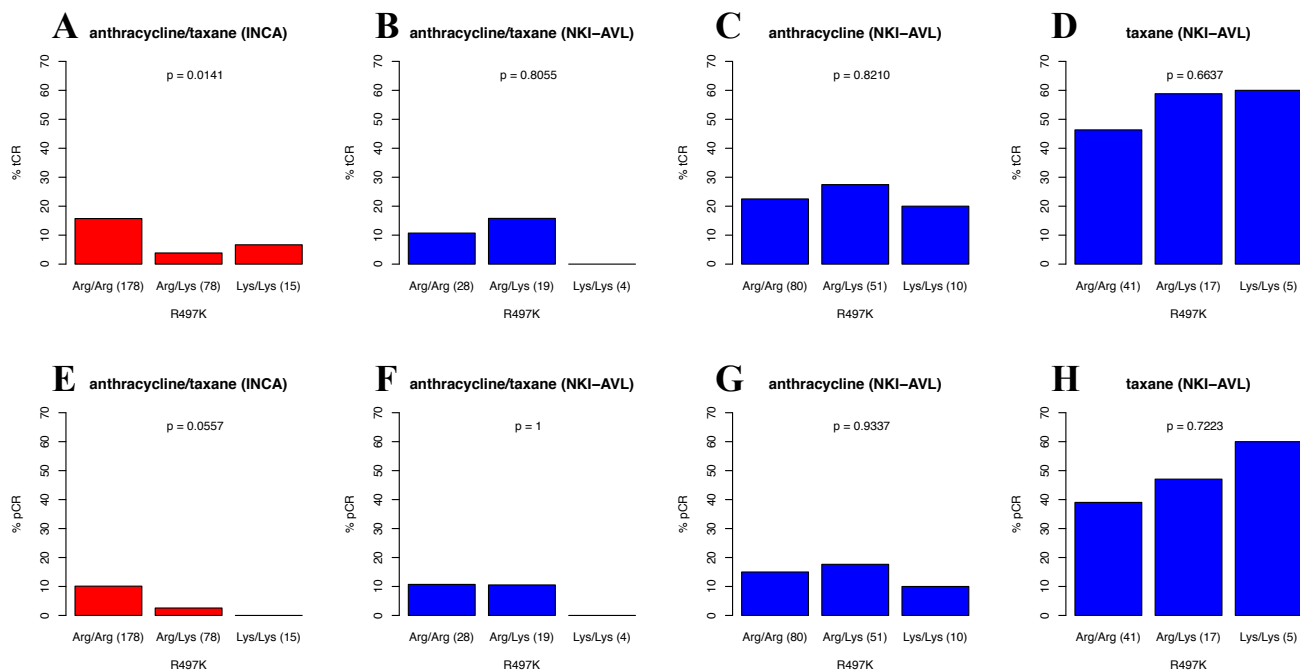

Supplement: S3 Fig — Proportions of cases who achieved tumor complete response (tCR) according to R497K genotypes after neoadjuvant treatment at INCA based on anthracycline/taxane chemotherapy protocols (A) or at NKI-AVL based on anthracycline/taxane (B), anthracycline (C) or taxane (D) protocols. In panels E-H, the same subgroup analysis comparing the proportion of pathological complete response status (pCR) at INCA (E) and NKI-AVL (F-H) cohorts. Numbers in parenthesis correspond to the total number of cases in each genotype group. P values were assessed by the Chi-square, or Fisher’s exact tests when counts were equal zero in at least one group. At the INCA cohort, the anthracycline/taxane protocol was majoritarian (94%) and, therefore, the sample size of other chemotherapy protocols was insufficient for comparisons. (PDF) [file pone.0189750.s005.pdf]
