# Supplementary material for: Evaluation of the EGFR polymorphism R497K in two cohorts of neoadjuvantly treated breast cancer patients
Source: PLoS One. 2017 Dec 21;12(12):e0189750. doi: 10.1371/journal.pone.0189750 (PMC5739423; doi:10.1371/journal.pone.0189750)
Supplement: S1 File — List of guidelines from the Recommendations for Tumor Marker Prognostic Studies (REMARK) that were used in this study. (PDF) [file pone.0189750.s006.pdf]

| | Item to be reported | Text / Table / Figure |
|---|---|---|
| **INTRODUCTION** | | |
| 1 | State the marker examined, the study objectives, and any pre-specified hypotheses. | 1- Introduction (last two paragraphs). |
| **MATERIALS AND METHODS** | | |
| *Patients* | | |
| 2 | Describe the characteristics (e.g., disease stage or co-morbidities) of the study patients, including their source and inclusion and exclusion criteria. | 2- Materials and Methods (Study population and references 25 and 29). |
| 3 | Describe treatments received and how chosen (e.g., randomized or rule-based). | 3- Materials and Methods (NAC protocols and reference 25 and 29). |
| *Specimen characteristics* | | |
| 4 | Describe type of biological material used (including control samples) and methods of preservation and storage. | 4- Materials and Methods (Genotyping). |
| *Assay methods* | | |
| 5 | Specify the assay method used and provide (or reference) a detailed protocol, including specific reagents or kits used, quality control procedures, reproducibility assessments, quantitation methods, and scoring and reporting protocols. Specify whether and how assays were performed blinded to the study endpoint. | 5- Material and methods: for clinical endpoint: (Clinical and Histopathological data); SNP: (Genotyping, supplementary figure 1 and references 11). |
| *Study design* | | |
| 6 | State the method of case selection, including whether prospective or retrospective and whether stratification or matching (e.g., by stage of disease or age) was used. Specify the time period from which cases were taken, the end of the follow-up period, and the median follow-up time. | 6- Material and methods (Study population, supplementary figure 2); also Statistical analyses (2nd paragraph). |
| 7 | Precisely define all clinical endpoints examined. | 7- Material and methods (Clinical and Histopathological data, last paragraph). |
| 8 | List all candidate variables initially examined or considered for inclusion in models. | 8- Supplementary table 1 and all the other tables (details described on each table footnote). Statistical analyses (2nd paragraph). |
| 9 | Give rationale for sample size; if the study was designed to detect a specified effect size, give the target power and effect size. | 9- Discussion (5th paragraph and supplementary figure 3). |
| *Statistical analysis methods* | | |
| 10 | Specify all statistical methods, including details of any variable selection procedures and other model-building issues, how model assumptions were verified, and how missing data were handled. | 10- Material and methods (Statistical analysis). Models and missing data were described in the text and detailed on each table footnote. |
| 11 | Clarify how marker values were handled in the analyses; if relevant, describe methods used for cutpoint determination. | 11- Allele grouping described |
| **RESULTS** | | |
| *Data* | | |
| 12 | Describe the flow of patients through the study, including the number of patients included in each stage of the analysis (a diagram may be helpful) and reasons for dropout. Specifically, both overall and for each subgroup extensively examined report the numbers of patients and the number of events. | 12- Supplementary figure 1. Number of events of each group is described in descriptive tables. |
| 13 | Report distributions of basic demographic characteristics (at least age and sex), standard (disease-specific) prognostic variables, and tumor marker, including numbers of missing values. | 13- Supplementary table 1 and table 1. |
| *Analysis and presentation* | | |
| 14 | Show the relation of the marker to standard prognostic variables. | 14- Table 2. |
| 15 | Present univariable analyses showing the relation between the marker and outcome, with the estimated effect (e.g., hazard ratio and survival probability). Preferably provide similar analyses for all other variables being analyzed. For the effect of a tumor marker on a time-to-event outcome, a Kaplan-Meier plot is recommended. | 15- Due the size of the tables, multivariable / adjusted ratios were demonstrated in table 3. Hazard ratio was described in table 4 and Kaplan-Meier curves in figure 1. |
| 16 | For key multivariable analyses, report estimated effects (e.g., hazard ratio) with | 16- Table 4. |

Source: McShane LM, Altman DG, Sauerbrei W, Taube SE, Gion M, Clark GM: Reporting recommendations for tumor marker prognostic studies (REMARK). *J Natl Cancer Inst* 2005; 97: 1180-1184.

| | | |
|---|---|---|
| | confidence intervals for the marker and, at least for the final model, all other variables in the model. | |
| 17 | Among reported results, provide estimated effects with confidence intervals from an analysis in which the marker and standard prognostic variables are included, regardless of their statistical significance. | 17- described in abstract and results section. |
| 18 | If done, report results of further investigations, such as checking assumptions, sensitivity analyses, and internal validation. | 18- Associations between neoadjuvant chemotherapy response and the marker were never described (in the best of our knowledge). |
| **DISCUSSION** | | |
| 19 | Interpret the results in the context of the pre-specified hypotheses and other relevant studies; include a discussion of limitations of the study. | 19- Results were interpreted on 3rd, 4th and 5th paragraphs of discussion. Limitations: last 5 paragraphs of the discussion. |
| 20 | Discuss implications for future research and clinical value. | 20- Last paragraph of the discussion and conclusion. |

Source: McShane LM, Altman DG, Sauerbrei W, Taube SE, Gion M, Clark GM: Reporting recommendations for tumor marker prognostic studies (REMARK). *J Natl Cancer Inst* 2005; 97: 1180-1184.
